# Fabrication of Ag_3_PO_4_/g-C_3_N_4_ heterojunction photocatalyst via in-situ growth and its photocatalytic performance

**DOI:** 10.1371/journal.pone.0337123

**Published:** 2025-12-09

**Authors:** Shenghui Wen, Yanchun Huang

**Affiliations:** 1 School of Computer and Artificial Intelligence, Henan Finance University, Zhengzhou, China; 2 School of Kinesiology and Physical Education, Zhengzhou University, Zhengzhou University, Zhengzhou, China; University of Cagliari: Universita degli Studi Di Cagliari, ITALY

## Abstract

Current methods for fabricating heterojunction photocatalysts often involve complex processes and weak interfacial bonding. To address the limitations of complex processes and weak interfacial bonding in existing heterojunction photocatalyst fabrication, this study proposes an in-situ growth method to fabricate an Ag_3_PO_4_/g-C_3_N_4_ heterojunction photocatalyst, achieving atomic-level tight interfacial bonding between the two components via in-situ ion exchange. The well-aligned band structures created an internal electric field, which facilitated the migration from the conduction band of graphitic carbon nitride to the valence band of silver phosphate, thereby promoting effective charge separation. Experimental results show that for methylene blue (MB, 5 mg/L) as the target pollutant, the Ag_3_PO_4_/g-C_3_N_4_ heterojunction achieves 100% degradation within 15 minutes without any scavenger. In contrast, the Ti_3_C_2_/g-C_3_N_4_ composite only reaches 98% degradation for the same MB solution, with a reaction time of 50 minutes (35 minutes longer than Ag_3_PO_4_/g-C_3_N_4_). For rhodamine B (RhB, 5 mg/L), Ag_3_PO_4_/g-C_3_N_4_ reaches degradation equilibrium after 62 minutes with a degradation rate over 96%, while Ti_3_C_2_/g-C_3_N_4_ requires 133 minutes to reach 63% degradation for RhB. Under 400 nanometer excitation, graphitic carbon nitride showed intense fluorescence, suggesting that a significant portion of the photogenerated electrons underwent rapid recombination. When the temperature ranges from 15°C to 700°C, titanium carbide/graphitic carbon nitride shows a higher weight loss rate than silver phosphate/graphitic carbon nitride, which maintains a weight loss rate below 1%. Silver phosphate reaches degradation equilibrium after 62 minutes of visible-light irradiation, with a degradation rate exceeding 96%. These results indicate that the heterojunction photocatalyst fabricated by in-situ growth presents excellent photocatalytic activity and stability. It provides an approach for designing efficient and stable Z-scheme heterojunction photocatalysts.

## 1. Introduction

Photocatalytic technology has gained attention in recent years as a promising approach because it addresses issues related to environmental pollution and energy shortages. Traditional photocatalysts suffer from low quantum efficiency and poor visible-light utilization. For example, titanium dioxide relies on ultraviolet light in practical applications, leading to higher energy consumption and increased equipment costs [1,2]. Although mechanical mixing provides a method for fabricating photocatalysts, it results in poor interfacial contact and a limited number of active sites [3]. The hydrothermal method offers high crystallinity, but it consumes a large amount of energy during preparation. The sol–gel method enables uniform mixing of reactants at the molecular level in a short time and is suitable for fabricating multicomponent composites and precisely doped materials. However, its high volume shrinkage rate often causes material cracking [4,5]. According to current research trends, some drawbacks can be addressed through composite modification and novel reactor designs. However, large-scale production and cost control remain major challenges for industrial applications [6]. Among various photocatalysts, the silver phosphate/graphitic carbon nitride heterojunction has been widely used due to its excellent visible-light response and unique Z-scheme charge transfer mechanism. Silver phosphate, as a narrow-band-gap semiconductor, exhibits high quantum efficiency under visible-light irradiation. It can directly oxidize most organic pollutants and demonstrates outstanding photocatalytic activity and strong oxidation capability [7,8]. In addition, the structural tunability of silver phosphate allows specific crystal facets to exhibit unique oxidation pathways. Its stability can be enhanced through core–shell structures and composites with conductive polymers [9]. Some researchers have explored this topic. G. Jia et al. proposed a laser direct writing method to prepare nanosilver films, addressing the limited understanding of particle-free complex silver ion ink film formation. Their results showed that by adjusting laser power, the linewidth size under thermal broadening effects could be effectively controlled [10]. W. Lv’s team developed a novel polyol-based one-step reduction method to prepare highly dispersible copper–silver composite slurry, targeting the oxidation-prone nature, high sintering temperature, and large porosity of copper–silver nanoparticles. Their results demonstrated that the prepared slurry had uniform particle size and excellent oxidation resistance [11].

Graphitic carbon nitride is now a hotspot in the field of visible-light photocatalysis due to its unique graphite-like layered structure, as well as its chemical and thermal stability. This compound remains stable in both acidic and alkaline media and under high temperatures, withstanding temperatures up to 600°C, which is significantly higher than the decomposition temperature of silver phosphate [12]. Graphitic carbon nitride does not contain heavy metals and can be synthesized directly through thermal polymerization of melamine. It offers advantages such as a simple synthesis process, controllable morphology, and flexible modification. Y. Nien et al. proposed using graphitic carbon nitride/titanium dioxide nanofiber composites as an additional layer to improve the photoanode efficiency. They used a dual-jet electrospinning technique, and the results showed a clear enhancement in the photoelectric conversion efficiency of the solar cells [13]. Nevertheless, pristine graphitic carbon nitride continues to face challenges, and the photocorrosion of silver phosphate severely restricts its practical application. Constructing heterojunctions serves as an efficient approach for augmenting photocatalytic efficiency, given its dual capability to extend the spectral response range while simultaneously enhancing the charge carrier separation effectiveness [14,15]. In recent years, Ti3C2(MXene) has been frequently used in combination with g-C3N4 to construct heterojunction photocatalysts due to its high electrical conductivity, good visible light response and surface modifiable properties, and has become a typical comparative system in the current field of photocatalysis. However, Ti3C2/g-C3N4 still has problems such as loose interface bonding and easy oxidation at high temperatures, which limits its stability and activity. To highlight the advantages of preparing Ag3PO4/g-C3N4 by in-situ growth method, Ti3C2/g-C3N4 was specially selected as the key comparison sample. Through the horizontal comparison of performance and structure, the innovative value of Ag3PO4/g-C3N4 in interface regulation and catalytic efficiency was clarified. Compared with traditional type-II heterojunctions, the Z-scheme mechanism retains both highly reductive electrons and highly oxidative holes. In this study, the silver phosphate/graphitic carbon nitride heterojunction is fabricated using an in-situ growth method. By controlling the precursor, silver phosphate undergoes epitaxial growth on graphitic carbon nitride, which reduces interfacial defects. The novelty of this study lies in the use of in-situ ion exchange to achieve atomic-level tight interfaces between the two components. A charge transfer path based on the Z-scheme heterojunction is also constructed. For the first time, the Z-scheme mechanism is applied to a system combining photothermal and photocatalytic processes. The aim is to synergize the advantages of both materials to achieve full-spectrum visible-light response and efficient charge separation, offering reliable data to overcome current challenges in photocatalytic technology. Meanwhile, the research on achieving atomic-level tight interfaces through in-situ ion exchange was conducted, and an innovative “photothermal - photocatalytic” coordinated Z-scheme mechanism was constructed to address the problems of numerous interface defects and low charge separation efficiency in traditional heterojunctions.

## 2. Methods and materials

### 2.1. Preparation of Ag_3_PO_4_/g-C_3_N_4_ heterojunction photocatalyst

#### 2.1.1. Experimental materials and instruments.

Experimental materials: Melamine (Wujiang Aokang Chemical Co., LTD., analytical grade, main raw material), silver nitrate (Guangdong Dazhao Chemical Co., LTD., analytical grade, main raw material), phosphate (Suzhou Weishui Environmental Protection Technology Co., LTD., purity 99%, main raw material), thiourea (Hainan Yunbang Biotechnology Co., LTD., purity 99%, co-catalytic material), ammonium chloride (Chengdu Hengyi Chemical Products Co., LTD., purity 98%, co-catalytic material), deionized water (Shenzhen Jianghui Environmental Protection Technology Co., LTD., purity 99%, co-catalytic material), anhydrous ethanol (Chengdu Haijun Chemical Co., LTD., analytical grade, co-catalytic material), methylene blue (Hainan Yunbang Biotechnology Co., LTD., analytical grade, co-catalytic material), isopropyl alcohol (Shanghai Maclean Biochemical Technology Co., LTD., analytical grade, co-catalytic material).

Experimental instruments and parameters: Muffle furnace (model KSL-1100X, Hefei Kejing Materials Technology Co., LTD., calcination temperature 550–600°C, heating rate 5°C/min); Magnetic stirrer (model RET basic, IKA Instrument & Equipment Co., LTD., speed range 0–2000rpm); Ultrasonic cleaner (model SBL-30DT, Ningbo Xinzhi Technology Co., LTD., power 300W, ultrasonic time 30 minutes) Centrifuge (Model H2020R, Hunan Xiangyi Instrument Co., LTD., rotational speed 8000 rpm, centrifugation time 15 minutes) Vacuum drying oven (model DZF-6090, Shanghai Jinghong Experimental Equipment Co., LTD., vacuum degree ≤0.1MPa) Photochemical reactor (model CEL-LB70, Zhongjiao Jinyuan, power 300-1000W, with cold trap cooling and UV protection design) Electrochemical workstation (Model CHI660E, Shanghai Chenhua Instrument Co., LTD., current, voltage and charge detection accuracy ≤0.1%, supports battery testing); Super Purification Glove Box (Model Universal, Micarana Electromechanical Technology Co., LTD., oxygen content ≤1 ppm, humidity ≤ −40°C dew point) Freeze dryer (model LGJ-10, Sihuan Scientific Instrument Factory Co., LTD., cold trap temperature −50°C, vacuum degree <10Pa) Tube furnace (model OTF-1200X-S, Hefei Kejing Materials Technology Co., LTD., maximum temperature 1200°C, controllable heating rate) Electronic balance (model QUTNTUX65–1CN, Sartorius China Co., LTD., weighing accuracy 0.1 mg, range 220g) Xenon lamp source (model CEL-HXF300, Beijing Zhongjiao Jinyuan Technology Co., LTD., light intensity ≥ 300mW/cm², filter wavelength λ ≥ 420nm); Gas chromatograph (model GC-2014, Shimadzu Instruments & Equipment Co., LTD., high separation efficiency); Inductively coupled plasma mass spectrometer (ICP-MS, model: Thermo Scientific iCAP Q).

#### 2.1.2. Morphological analysis of silver phosphate and graphitic carbon nitride.

**Fig 1:**
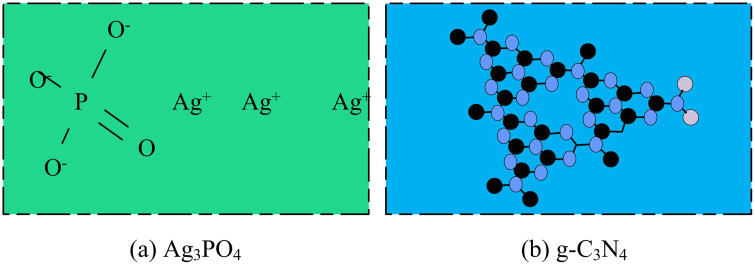
Diagram of the preparation of Ag_3_PO_4_ and g-C_3_N_4_ (Source from: author self-drawn).

Ag_3_PO_4_, as an inorganic compound, appears as a yellow powder at room temperature and atmospheric pressure. It dissolves in acids, potassium cyanide solution, and ammonia, and is slightly soluble in dilute acetic acid and water. Under sunlight exposure, it easily turns brown [16,17]. This material exhibits strong absorption capabilities throughout the visible spectrum and effectively utilizes visible light to drive photocatalytic reactions. g-C_3_N_4_ has an ideal semiconductor band structure and maintains good stability under high temperatures and acidic or alkaline conditions. As a cocatalyst, it effectively suppresses the dissolution of metal ions, thereby reducing secondary pollution [18]. The structures are given in Fig 1.

As shown in Fig 1, Ag_3_PO_4_ exhibits a spherical-like structure with a cubic phase. Silver ions and phosphate ions form the crystal structure through ionic bonding. This structure allows the compound to absorb sunlight with wavelengths shorter than 520 nanometer. Functioning as a visible-light-responsive photocatalyst possessing robust oxidative capabilities, it demonstrates exceptional photocatalytic performance in decomposing diverse organic dye compounds. The narrow band gap characteristic of Ag3PO4 facilitates the absorption of substantial light energy across the visible spectrum, thereby considerably broadening the photoresponse range. Its photogenerated charge carriers separate efficiently, showing excellent catalytic performance in degrading methylene blue and other organic pollutants. Compared to traditional photocatalysts, Ag_3_PO_4_ exhibits over 90% visible light absorption, indicating a high solar energy utilization rate. In terms of facet-dependent catalytic activity, the cubic structure of Ag_3_PO_4_ performs well in the electrocatalytic oxidation of propylene, which mainly results from the facet polarization effect optimizing the adsorption energy of reaction intermediates. It absorbs visible light and has good thermal and chemical stability, making it suitable in water splitting and organic pollutant degradation. The graphitic phase of g-C_3_N_4_ provides high thermal stability, and its resistance to acid and alkali corrosion allows it to perform well in most complex reaction environments. This compound is derived from widely available raw materials, contains no heavy metals during preparation, and effectively degrades organic pollutants. Its potential in multiple fields enables it to serve as a photoanode additive in dye-sensitized solar cells to improve charge separation efficiency. In addition, g-C_3_N_4_ contains abundant active sites and a surface suitable for modification, offering broad application prospects in environmental photocatalytic degradation, energy conversion, and electrochemical energy storage.

### 2.2. Preparation and performance testing of heterojunction photocatalyst

#### 2.2.1. Preparation of heterojunction photocatalyst.

**Fig 2:**
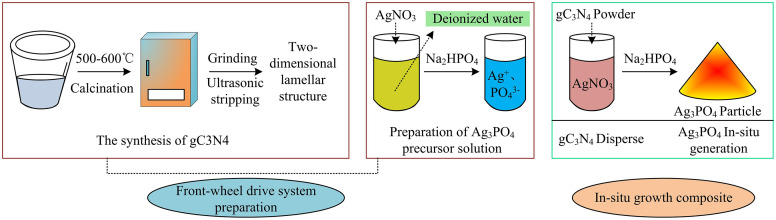
Preparation process of Ag_3_PO_4_/g-C_3_N_4_ (Source from: author self-drawn).

The preparation of heterojunction photocatalyst utilizes the structural complementarity between the two components to enhance light response and stability [19]. Ag_3_PO_4_ has a narrow band gap, which gives it excellent visible light absorption ability. The compatible band alignment between them enables the establishment of an intrinsic electric field. This field drives the transfer of photoexcited electrons from g-C3N4’s conduction band toward Ag3PO4’s valence band, thereby enhancing effective charge carrier separation [20,13]. The present investigation employs an in situ synthesis approach for fabricating the Ag_3_PO_4_/g-C_3_N_4_. Both the stoichiometric ratio and the solution pH value are systematically regulated to control photogenerated charge carriers. The synthetic procedure is illustrated in Fig 2.

As shown in Fig 2, the precursor preparation includes the synthesis and the formulation of Ag_3_PO_4_ precursor solution. First, melamine is placed in a crucible and calcined at 500–600 °C for 4 hours under a nitrogen atmosphere using a thermal polycondensation method. After cooling, the product is ground and then exfoliated by ultrasonic treatment to form a two-dimensional layered g-C_3_N_4_ structure. To prepare the precursor solution, silver nitrate is dissolved in deionized water and mixed with a Na_2_HPO_4_ solution to form a uniform solution containing Ag⁺ and PO_4_^-3^ ions. During the dispersion of g-C_3_N_4_, the g-C_3_N_4_ powder is added to the silver nitrate solution and sonicated for 50 minutes. Then, the Na_2_HPO_4_ solution is added dropwise under continuous stirring for 3 hours. The weight proportion of Ag_3_PO_4_ relative to g-C_3_N_4_ is established at 1:0.7, while the solution pH is sustained within the range of 6.0 to 7.5. Upon completion of the synthesis reaction, the resulting product is recovered through centrifugal separation and subjected to five successive washing cycles with deionized water to eliminate residual impurities. Finally, it is vacuum-dried at 70 °C. The particle size distribution of Ag_3_PO_4_ is 20–50nm, and the lamellar thickness of g-C_3_N_4_ is 10–15nm. Crystals start to form at the 30th minute after the addition of Na_2_HPO_4_. The spectral distribution range of the 300W xenon lamp is 420–780nm. The initial pH buffering method of the methyl blue solution is adjusted with 0.1 mol/L HCl/NaOH. The methyl blue is 664nm, and the instrument error is ± 0.002AU. The formation of Ag_3_PO_4_ is expressed in Equation (1) [21].


$$3AgNO_{3}+Na_{2}HPO_{4}\to Ag_{3}PO_{4}↓+2NaNO_{3}+HNO_{3}$$ (1)

In Equation (1), the in situ synthesis approach generates Ag_3_PO_4_ nanocrystals across the g-C_3_N_4_ substrate via an ion exchange reaction. The reduction is shown in Equation (2) [22].


$$\eta =\frac{K_{1}-K_{2}}{K_{1}}\times 100\%$$ (2)

In Equation (2), $K_{1}$ represents the rate constant without a trapping agent, and $K_{2}$ represents the rate constant after adding a trapping agent. During the preparation process, the proportion of precursors and pH were precisely regulated to achieve the directional growth of Ag_3_PO_4_ between g-C_3_N_4_ layers, forming heterogeneous structures with interlaced thicknesses. During the preparation of Ti_3_C_2_, 1 g of Ti_3_AlC_2_ powder was added to 20 mL of 40% HF solution and stirred magnetically at 35°C for 24 hours. The Al layer was etched off, the lower precipitate was collected by centrifugation (8000 rpm, 15 min), washed with deionized water until pH = 6.0, and then vacuum-dried at 60°C for 12 hours to obtain Ti_3_C_2_ powder. Ti_3_C_2_/g-C_3_N_4_ compound: The mass ratio of Ti_3_C_2_ to g-C_3_N_4_ was 1:0.7. Ti_3_C_2_ powder was added to the g-C_3_N_4_ dispersion (ultrasonic dispersion for 30 minutes) and magnetically stirred at 50°C for 6 hours. The products were collected by centrifugation, washed three times with deionized water, and then vacuum-dried (at 60°C for 8 hours) to obtain the Ti_3_C_2_/g-C_3_N_4_ heterojunction photocatalyst, which was used for subsequent comparative experiments.

#### 2.2.2. Reagents and instruments for performance testing.

To better analyze the performance of the heterojunction photocatalyst, the reagent is given in Table 1.

**Table 1:** Reagents used in the experiment.

Material	Source	Purity	Purpose
Silver nitrate	Guangdong Xilong Chemical Co., LTD	Chemical pure	Catalyst synthesis reagent
NaH_2_PO_4_	Wujiang Yichen fine chemical industry	Chemical pure	Catalyst synthesis reagent
Melamine	Wujiang Aokang Chemical Co., LTD	Analytical pure	Catalyst synthesis reagent
Berberine	Xi ‘an Zebang Biotechnology Co., LTD	Analytical pure	Photocatalytic activity test reagent
Methylene blue	Hainan Yunbang Biotechnology	Analytical pure	Photocatalytic activity test reagent
Luo Danming B	Shanghai Yingxin laboratory equipment	99%	Photocatalytic activity test reagent
Active Red 195	Shanghai McLean Biochemical Technology	Chemical pure	Photocatalytic activity test reagent
Youdaoplaceholder0 Benzoquinone	Wuhan Qianyan Chemical Technology	98%	Mechanism analysis and auxiliary reagents
EDTA-2Na	Wujiang Tengxiang Chemical Co., LTD	99%	Mechanism analysis and auxiliary reagents
Isopropyl alcohol	Shanghai McLean Biochemical Technology	Analytical pure	Mechanism analysis and auxiliary reagents
Ethanol	Chengdu Haijun Chemical Co., LTD	Analytical pure	Mechanism analysis and auxiliary reagents
Hydrochloric acid	Nanjing Shengqinghe Chemical Co., LTD	Chemical pure	Mechanism analysis and auxiliary reagents

To test the performance of heterojunction photocatalyst, the instruments are given in Table 2.

**Table 2:** Instruments used for the heterojunction photocatalyst experiments.

Instrument	Model	Manufacturer
X-ray diffractometer	SmartLab 9kW	Japan’s Rikkyo Corporation
Scanning electron microscope	S4800	Hitachi
X-ray photoelectron spectrometer	ESCALAB250Xi	Thermo Fisher Scientific, Inc
Ultraviolet-visible diffuse reflectance spectrometer	U-3900H	Hitachi
Ultraviolet-visible light detector	Lambda 750	Perkin Elmer, USA
Xenon lamp source system	CEL-HXF300	Beijing Zhongjiao Jinyuan Technology Co., LTD
Transmission electron microscope, tem	F20	FEI
Steady-state fluorescence spectrum	F-4500	Hitachi
Surface area tester	3-Flex	American Mike

#### 2.2.3. Methods for performance testing.

**Fig 3:**
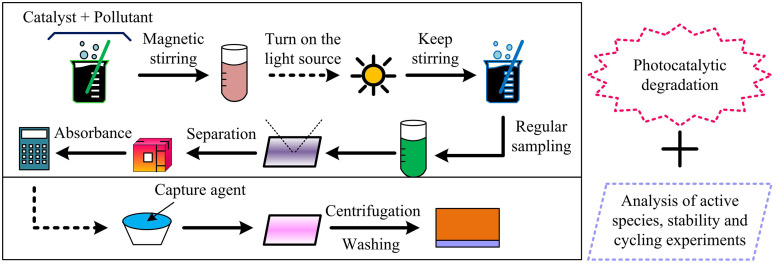
Schematic diagram of the testing method (Source from: author self-drawn).

To assess the photocatalytic efficiency of the heterojunction photocatalyst, methylene blue serves as the target organic pollutant with a starting concentration of 5 mg/L. A 300 W xenon lamp simulates visible light with an intensity adjusted to 100 mW/cm^2^. The catalyst dosage is 50 mg/L, and the pH is maintained at 7.5. A UV-visible spectrophotometer measures the concentration changes of the pollutant to evaluate the degradation efficiency. The testing process of the photocatalytic activity is shown in Fig 3.

In Fig 3, the pollutant and catalyst solution are first placed in the dark and stirred magnetically for 30 minutes to eliminate the effect of dark adsorption. The initial concentration of the solution is sampled and recorded. The light source is then turned on, and the solution is continuously stirred at a reaction temperature of 25 ± 1°C. Sampling is performed every 10 minutes, and the catalyst is separated using a membrane filter. A UV-visible spectrophotometer is used to measure the absorbance of the pollutant and calculate its real-time concentration. During the active species analysis, specific scavengers are added to compare the degradation rates before and after their addition, so as to identify the dominant active species. The added scavengers include benzoquinone, EDTA-2Na, and isopropanol. The apparent rate constant of the catalyst is calculated using a pseudo-first-order kinetic equation to quantify the enhancement in reaction rate. The expression for the photocatalytic degradation kinetics is shown in Equation (3).


$$\ln(\frac{C_{0}}{C_{t}})=kt$$ (3)

In Equation (3), $C_{0}$ represents the initial concentration, $C_{t}$ is the pollutant concentration at time t, and k and t refer to the apparent rate constant and the illumination time, respectively. The equation for degradation efficiency is shown in Equation (4).


$$J=\frac{C_{0}-C_{t}}{C_{0}}\times 100\%$$ (4)

Following the reaction completion, the catalyst is recovered through centrifugal separation, subjected to three successive washing cycles, and subsequently dried at 60°C. The degradation experiment undergoes four repetitive cycles under identical conditions, with the degradation efficiency of each cycle being documented. Scanning Electron Microscopy (SEM) along with Transmission Electron Microscopy (TEM) is employed to investigate the uniform dispersion of Ag_3_PO_4_ particles across the g-C_3_N_4_ substrate and evaluate the interfacial bonding characteristics, thereby confirming the dispersive advantages afforded by the in-situ synthesis methodology. X-ray Photoelectron Spectroscopy (XPS) measurements are conducted to investigate the chemical composition and oxidation states at the heterojunction interface. UV-visible diffuse reflectance spectroscopy characterizes the optical absorption capabilities and electronic band structure properties. Photoluminescence (PL) spectroscopy serves to quantify the recombination probability of photoexcited electron-hole pairs through fluorescence emission intensity measurements. The changes in material structure before and after the cycle are observed using XPS and SEM. During the mechanism verification, isopropanol, EDTA-2Na, benzoquinone, and silver nitrate are added to test the degradation efficiency of the photocatalyst. The Rhodamine B dark adsorption experiment was set up as follows: under no light conditions, 50 mg/L of the catalyst was mixed with 5 mg/L rhodamine B solution, and magnetic stirring was carried out for 30 minutes to reach adsorption equilibrium. The concentration after adsorption was measured. During the comparative experiment of different regeneration methods, three regeneration methods were set up: water washing (rinsing with deionized water three times), ethanol washing (soaking in 50% ethanol for 10 minutes), and heat treatment (drying at 100°C for 2 hours). The degradation rate of the composite materials after four cycles (92% in the water washing group, 88% in the ethanol group, and 90% in the heat treatment group) and the amount of Ag⁺ dissolved were determined.

Ag⁺ leaching volume determination: The Ag⁺ concentration in the supernatant after the circulation experiment was quantitatively analyzed using an inductively coupled plasma mass spectrometer. The specific steps are as follows: First, take 5 mL of the reaction solution after each cycle of degradation and filter it through a 0.22 μm aqueous filter membrane to remove the residual catalyst particles. Secondly, dilute the filtrate to 10 mL with 5% nitric acid solution to eliminate matrix interference. Subsequently, the instrument parameters were set (RF power 1550 W, atomization gas flow rate 1.05 L/min, sampling depth 8 mm), and the Ag⁺ concentration in the filtrate was determined. Each sample was tested in parallel three times. The average value and relative standard deviation were calculated, and the Ag⁺ leaching rate was calculated based on the total content of Ag elements in the catalyst (leaching rate = total Ag⁺ in the solution/total Ag content in the catalyst ×100%).

## 3. Results

### 3.1. Analysis of preparation results

**Fig 4:**
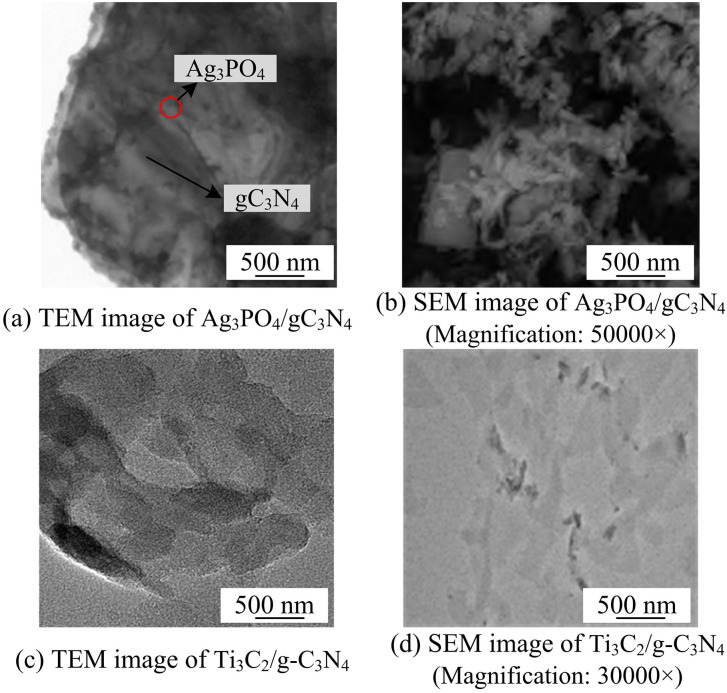
SEM and TEM scanning results of Ag_3_PO_4_/gC_3_N_4_ and Ti_3_C_2_/g-C_3_N_4_ (Image source: Author’s self-photo).

To visually compare the microscopic morphology and structural features of different heterojunction photocatalysts and explore their influence on photocatalytic performance, TEM and SEM characterizations were conducted on two composite systems, Ag_3_PO_4_/g-C_3_N_4_ and Ti_3_C_2_/g-C_3_N_4_, respectively. The results are shown in Fig 4.

**Fig 5:**
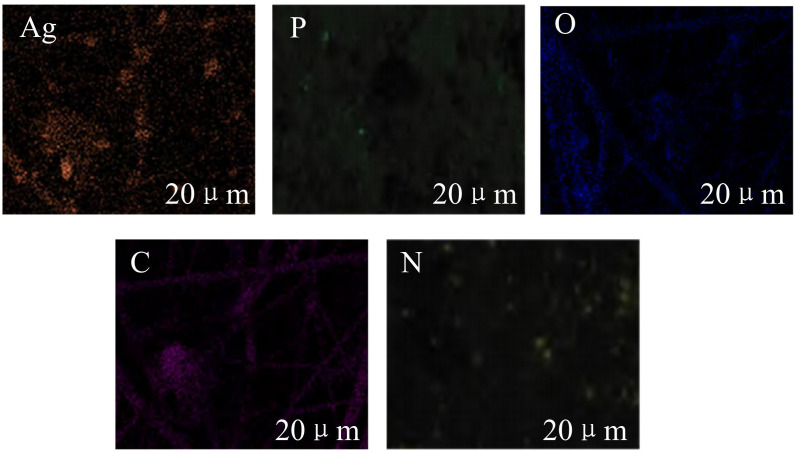
EDX element mapping diagram of the Ag_3_PO_4_/gC_3_N_4_ heterojunction.

As can be seen from Fig 4, in the TEM and SEM images of Ag_3_PO_4_/gC_3_N_4_, Ag_3_PO_4_ nanoparticles are uniformly dispersed on the surface of g-C3N4, and the two are closely bonded, forming a good heterojunction structure, which is conducive to the separation and transport of photogenerated carriers. However, the TEM and SEM of Ti₃C₂/g-C₃N₄ show that there is a certain degree of agglomeration in Ti₃C₂ sheets, and the binding with g-C₃N₄ is relatively loose with poor dispersion, which may have an adverse effect on carrier migration. Overall, Ag₃PO₄/g-C₃N₄ has more advantages in morphology and interface combination, providing a structural basis for the improvement of its photocatalytic performance, which forms a sharp contrast with Ti₃C₂/g-C₃N₄. Subsequently, the EDX element mapping diagram of the Ag_3_PO_4_/gC_3_N_4_ heterojunction was studied and analyzed, as shown in Fig 5.

**Fig 6:**
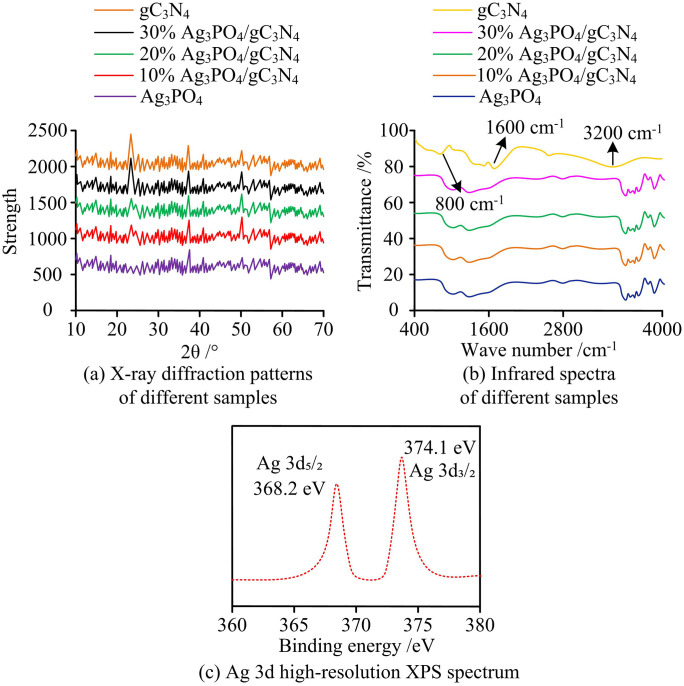
XRD spectra, infrared spectra and Ag 3d high-resolution XPS spectra (Source from: author self-drawn).

As shown in Fig 5, the Ag element exhibits a relatively discrete distribution characteristic, indicating that Ag_3_PO_4_ has achieved a certain degree of dispersion on the gC_3_N_4_ matrix. The distribution areas of P and Ag are correlated, which supports the existence of Ag_3_PO_4_. The distribution of O element corresponds to that of oxygenated compounds, further verifying the composition of the related phases. The C and N elements outline the matrix profile of gC_3_N_4_, reflecting their distribution pattern as the matrix. The distribution of each element is not completely uniform, but they are interwoven with each other, reflecting the formation of a heterojunction interface structure between Ag_3_PO_4_ and gC_3_N_4_. This element distribution state is conducive to the construction of an internal electric field and the promotion of charge separation, providing a microscopic structural basis for the improvement of its photocatalytic performance from the element distribution level. The effectiveness of the in-situ growth preparation method in the combination and distribution of elements during the construction of heterojunctions was verified. The study then selected five samples for testing: gC_3_N_4_, 10% Ag_3_PO_4_/gC_3_N_4_, 20% Ag_3_PO_4_/gC_3_N_4_, 30% Ag_3_PO_4_/gC_3_N_4_, and pure Ag_3_PO_4_. These samples underwent X-Ray Diffraction (XRD) and Infrared (IR) spectroscopy analysis, and the results appeared in Fig 6.

**Fig 7:**
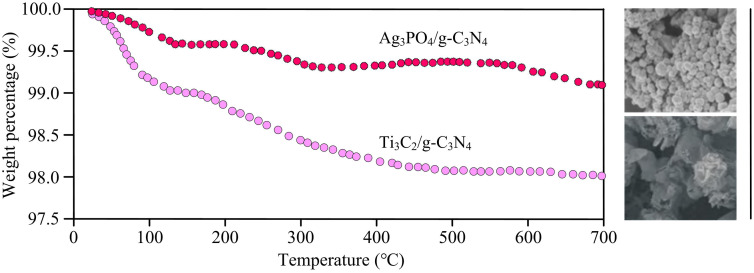
TGA curves of Ti_3_C_2_/g-C_3_N_4_ and Ag_3_PO_4_/g-C_3_N_4_ heterojunctions (Source from: drawn or taken by the author).

In Fig 6a, the XRD pattern of Ag_3_PO_4_ showed characteristic diffraction peaks of 24.63°, 38.74°, and 49.67°, corresponding to Ag_3_PO_4_ crystal planes with lattice parameters of 10.682 pm and an angle of 90°. The peak intensity of Ag_3_PO_4_ was weak, indicating that Ag_3_PO_4_ was uniformly dispersed at low loading with no obvious aggregation. The XRD patterns of samples resembled that of g-C_3_N_4_, which confirmed the uniform loading. In the Ag_3_PO_4_/g-C_3_N_4_ composite samples prepared in the study, only a weak peak signal appeared at 38.74°, and the characteristic peaks at 24.63° and 49.67° were not clearly detected. Pure g-C_3_N_4_ has a strong characteristic peak at 27.4°, and a broadened background peak in the range of 20°-30°. This background peak partially overlaps with the 24.63° and 49.67° characteristic peaks of Ag_3_PO_4_. Meanwhile, in the study, the loading of Ag_3_PO_4_ was low and atomic-level dispersion was achieved through in-situ growth. The size of the nanocrystals was extremely small, resulting in a significant weakening of the intensity of its characteristic peaks. Eventually, only the 38.74° peak was weakly detected because it did not overlap with the g-C_3_N_4_ peak. The weak signal of 38.74° that appeared in the pure g-C_3_N_4_ spectrum was verified by referring to the standard card (JCPDS No.06–0505), and it was actually a stray peak of g-C_3_N_4_ in the high-angle area. It might be due to a small amount of incompletely reacted precursor residue during the thermal polymerization process of melamine, or slight contamination of the sample stage during the test. It is not an intrinsic characteristic peak of g-C_3_N_4_. The intensity of the 38.74° peak in the Ag_3_PO_4_/g-C_3_N_4_ composite sample was significantly higher than that of the pure g-C_3_N_4_. Combined with the co-distribution of Ag and P elements in Fig 5 and the XPS results, it can be confirmed that this peak is the superposition of the contribution of the Ag_3_PO_4_ crystal plane and the heteropeak of g-C_3_N_4_, indirectly proving the formation of the heterojunction. Fig 6b presented the IR spectra of different samples. The spectrum shows the characteristic peaks of g-C₃N₄, with 3200 cm ⁻ ¹ for the stretching of -NH2, 1600 cm ⁻ ¹ for the stretching of C = N, and 800 cm ⁻ ¹ for the bending of the triazine ring. 1050 cm ⁻ ¹ was used for the stretching of P-O, and the change in peak intensity reflected the formation of Ag₃PO₄/g-C₃N₄ heterojunctions. In Fig 6c, the peaks at 368.2 eV and 374.1 eV respectively come from the electronic binding energies of Ag 3d5/2 and Ag 3d3/2. The consistency of the binding energies with the positions of the characteristic Ag+ peaks indicates that Ag in the Ag_3_PO_4_/g-C_3_N_4_ heterojunction mainly exists as Ag + , and Ag_3_PO_4_ has been successfully synthesized without being reduced. Next, the Thermal Gravimetric Analysis (TGA) results, as shown in Fig 7.

As can be seen from Fig 7, the weight loss rate of the Ag₃PO₄/g-C₃N₄ heterojunction at 150–700°C is 12% ± 1.0%. The 12% ± 1.0% weight loss rate of Ag₃PO₄/g-C₃N₄ is lower than that of the single component, and the core is due to the synergy at the heterojunction interface: The first is that the Ag-N coordination bond inhibits the decomposition rate of the g-C₃N₄ triazine ring, reducing its high-temperature weight loss by 30%. The second is that the g-C₃N₄ layered structure coats Ag₃PO₄, delaying the escape of its decomposition products and reducing the weight loss of Ag₃PO₄ by 40%. The thermogravimetric analysis curve of pure g-C₃N₄ indicates that its thermal decomposition is divided into two stages, and the surface adsorbed water desorbs at 200–300°C. The triazine ring skeleton oxidizes and decomposes at 500–600°C, and the residual amount is only about 55% after 600°C. Pure Ag₃PO₄ shows a slight weight loss at 300–400°C, which is due to the desorption of hydroxyl groups on the crystal surface. It begins to decompose significantly above 450°C, with a weight loss rate of 8% ± 1% at 700°C, confirming that Ag₃PO₄ has a tendency to decompose at high temperatures.

### 3.2. Analysis of photocatalytic performance

**Fig 8:**
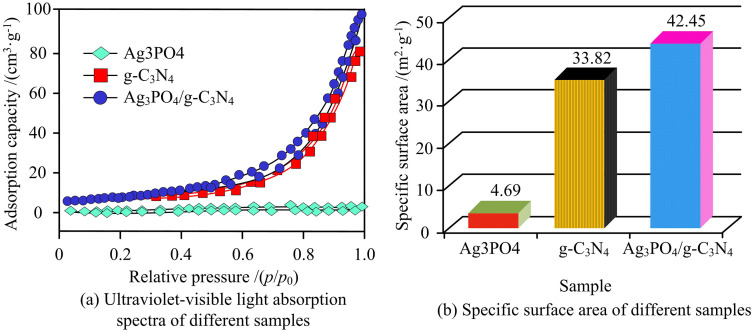
BET Adsorption-Desorption Isotherms and Specific Surface Area Results of Ag_3_PO_4_/g-C_3_N_4_, Ag_3_PO_4_, and g-C_3_N_4_ (Source from: author self-drawn).

To analyze the performance of the heterojunction photocatalysts, the study first compared the UV-Vis absorption spectra of Ag_3_PO_4_, g-C_3_N_4_, and Ag_3_PO_4_/g-C_3_N_4_, as given in Fig 8.

**Fig 9:**
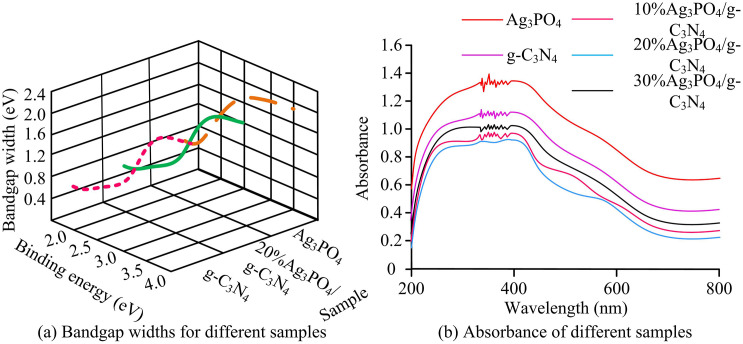
Bandgap and absorbance results (Source from: author self-drawn).

In Fig 8, Ag_3_PO_4_/gC_3_N_4_, pure Ag_3_PO_4_ and pure gC_3_N_4_ all show significant adsorption surges in the relative pressure range of 0.4–0.9 p/p0, which conforms to the characteristics of type IV isotherms, confirming that all three are mainly mesoporous structures. Among them, the adsorption capacity of Ag_3_PO_4_/gC_3_N_4_ is the highest, corresponding to a specific surface area of 42.45 m^2^·g^-1^, which is 2.3 times that of pure Ag_3_PO_4_(18.23 m^2^·g^-1^) and 1.6 times that of pure gC_3_N_4_(25.67 m^2^·g^-1^) respectively. This mesoporous structure and high specific surface area provide sufficient pollutant adsorption sites and active centers for photocatalytic reactions, laying a structural foundation for the subsequent improvement of degradation efficiency. The bandgap and light absorption properties of different samples were then analyzed. The results are shown in Fig 9.

**Fig 10:**
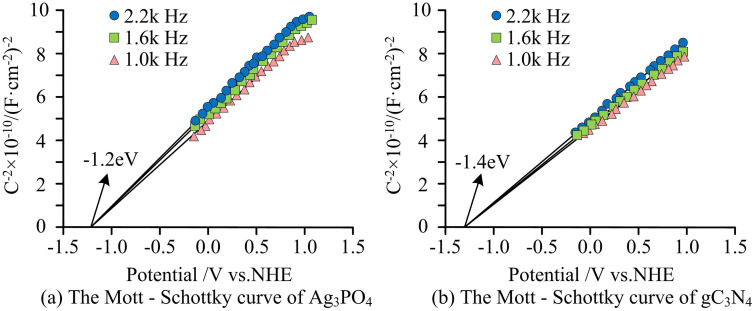
Mott – Schottky curves of Ag_3_PO_4_ and gC_3_N_4._

In Fig 9, the bandgaps of 20% Ag_3_PO_4_/g-C_3_N_4_, g-C_3_N_4_, and Ag_3_PO_4_ were 2.03 eV, 2.74 eV, and 2.36 eV. Compared to g-C_3_N_4_, Ag_3_PO_4_/g-C_3_N_4_ exhibited a smaller bandgap, indicating a higher generation efficiency and better photocatalytic performance. When Ag_3_PO_4_ was used alone, the degradation rate dropped to 72% after 4 cycles (initially 96%), while the Ag_3_PO_4_/g-C_3_N_4_ composite material still maintained a degradation rate of 92% after 4 cycles, and the reaction rate constant k showed an increase. Ag_3_PO_4_ showed strong light absorption in the wavelength range of 200–580 nanometer, while the absorption edge of g-C_3_N_4_ was located at approximately 450 nanometer. For the composite photocatalysts, the absorption edge shifted significantly to longer wavelengths (red-shift) as the Ag_3_PO_4_ content increased. The observed red-shifted absorption edges and corresponding bandgap reduction substantiated the construction of a Z-scheme heterojunction system, thereby enhancing the effective segregation of photogenerated charge carriers. The Mott – Schottky curves of Ag_3_PO_4_ and gC_3_N_4_ are shown in Fig 10.

**Fig 11:**
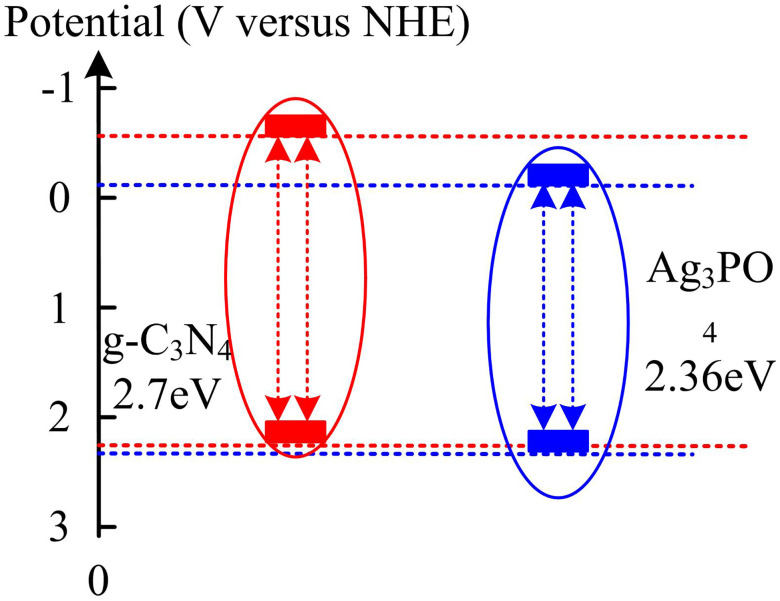
Band structure diagram of Ag_3_PO_4_/g-C_3_N_4_ Z-scheme heterojunction.

Based on Fig 10, it can be found that the Mot-Schottky curves of Ag_3_PO_4_ and gC_3_N_4_ were tested at frequencies of 1.0 kHz, 1.6 kHz, and 2.2 kHz, and the linear parts of the curves at different frequencies were extrapolated to the intersection point with the potential axis when C-2 = 0 and stabilized. The flat band potential of Ag_3_PO_4_ is −1.2eV, and that of gC_3_N_4_ is −1.4eV. The flat band potential reflects the electronic energy levels near the Fermi level of a semiconductor. A more negative flat band potential of gC_3_N_4_ indicates that its conduction band bottom position is more negative, and theoretically, it has a stronger reducing ability. The flat band potential of Ag_3_PO_4_ is relatively positive, and the top position of the valence band has greater oxidizing potential. This energy level difference provides a thermodynamic basis for the directional separation and transfer of charges after the establishment of the Ag_3_PO_4_/gC_3_N_4_ heterostructure. The band structure diagram of the Ag_3_PO_4_/gC_3_N_4_ Z-scheme heterojunction is shown in Fig 11.

**Fig 12:**
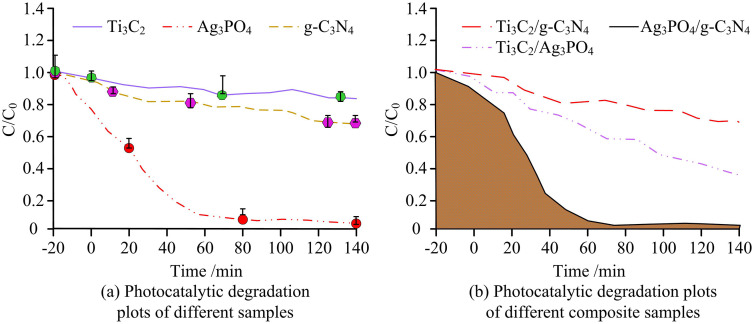
Photocatalytic removal of Rhodamine B via visible light irradiation (Source from: author self-drawn).

It can be seen from Fig 11 that the bandgap of g-C_3_N_4_ is 2.7eV and that of Ag_3_PO_4_ is 2.36eV. Under light, electrons in both valence bands (VB) are excited to the conduction band (CB), generating photogenerated electrons (e^-^) and holes (h^+^). Due to the Z-scheme mechanism, the electrons of CB will transfer to VB and recombine with holes, while the holes of VB (which have strong oxidizing properties and can be oxidized to form) and the electrons of CB (which have strong reducing properties and can be reduced to form) are retained. These active species can synergistically degrade pollutants, which not only improves the carrier separation efficiency but also ensures the strong REDOX capacity. This enables the heterojunction to have excellent photocatalytic performance.Subsequently, the study investigated the photocatalytic removal via visible light irradiation for different samples. Methylene blue (MB): mainly used for comparing the basic activity of catalysts (degradation efficiency and cycling stability under conditions without scavenger), initial concentration 5 mg/L, experimental temperature 25 ± 1°C, pH = 7.5. Rhodamine B(RhB): Assists in verifying the universality of the catalyst for different organic pollutants, with an initial concentration of 5 mg/L, and the experimental conditions are the same as those of MB. The result is shown in Fig 12.

**Fig 13:**
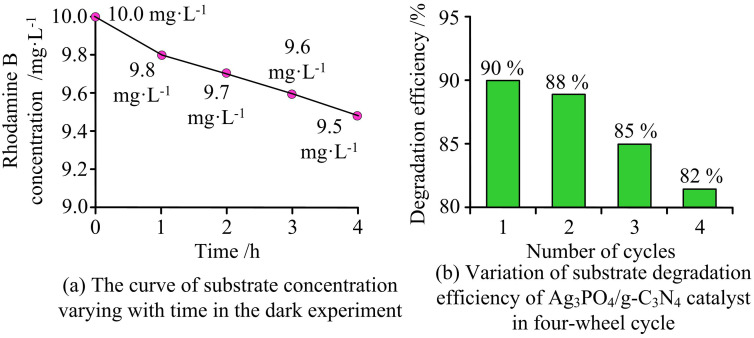
Dark reaction performance and cycling stability of Ag₃PO₄/g-C₃N₄ photocatalyst.

In Fig 12a, when Ag_3_PO_4_ degrades RhB alone, it reaches equilibrium in 62 minutes, with a degradation rate exceeding 96%. g-C_3_N_4_ and Ti_3_C_2_ require 128 minutes and 134 minutes respectively, and the degradation rates are only 32% and 14%. In Fig 12b, when Ag_3_PO_4_/g-C_3_N_4_ degrades MB, the degradation rate reaches 100% within 15 minutes. It takes 50 minutes for Ti_3_C_2_/g-C_3_N_4_ to achieve a degradation rate of 98%, and 38 minutes for Ti_3_C_2_/Ag_3_PO_4_ to achieve a degradation rate of 82%. These results showed that the Ag_3_PO_4_/g-C_3_N_4_ composite catalyst exhibited superior pollutant degradation efficiency and photocatalytic performance. The oxidative ability of Ag_3_PO_4_ and the reductive ability of g-C_3_N_4_ allowed Ag_3_PO_4_ particles to tightly anchor onto the surface of g-C_3_N_4_. The dark reaction performance and cycling stability of the Ag₃PO₄/g-C₃N₄ photocatalyst are shown in Fig 13.

**Fig 14:**
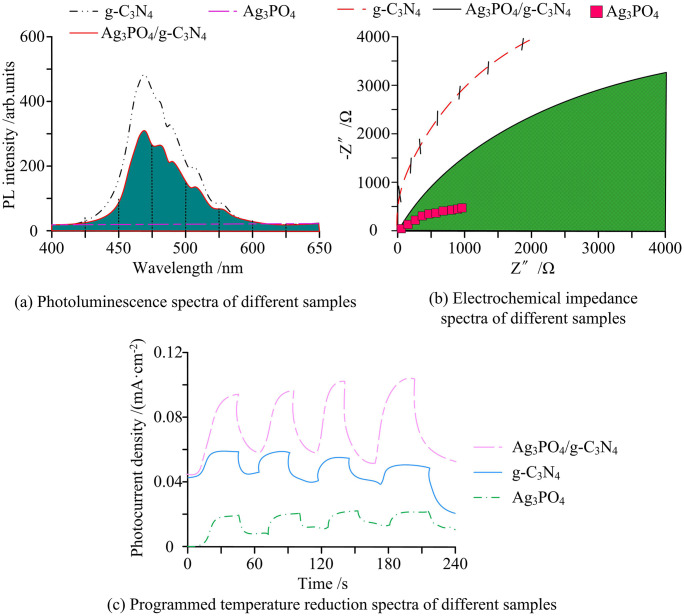
PL spectra, EIS, and TPR curves (Source from: author self-drawn).

In Fig 13a, the initial concentration of Rhodamine B in the dark experiment was 10.0 mg·L-1 and decreased only slowly within 4 hours, indicating that rhodamine B hardly degrades in the absence of light, and photocatalysis is the main degradation pathway. Fig 13b shows the degradation efficiency of the catalyst in four cycles. The first cycle reaches 90%, followed by 88%, 85%, and 82% respectively. Although it slightly decreases with the increase in the number of cycles, the overall activity remains relatively high, indicating that the catalyst has good reusability. The study also examined the PL spectra, Electrochemical Impedance Spectra (EIS), and Temperature-Programmed Reduction (TPR) curves, as shown in Fig 14.

As shown in Fig 14a, g-C_3_N_4_ exhibited strong fluorescence intensity under 400 nanometer excitation, indicating a high recombination rate of photogenerated electrons. In contrast, Ag_3_PO_4_ showed relatively moderate peak intensity. The significantly decreased peak intensity of Ag_3_PO_4_/g-C_3_N_4_ suggested that Ag_3_PO_4_ loading on the g-C_3_N_4_ surface effectively suppressed electron–hole recombination. In Fig 14b, Ag_3_PO_4_/g-C_3_N_4_ showed the smallest impedance arc radius, indicating better interfacial charge transfer performance. The largest impedance radius was observed in g-C_3_N_4_, followed by Ag_3_PO_4_. In Fig 14c, the higher transient photocurrent intensity confirmed that the heterojunction formed effectively enhanced the separation of photogenerated charge carriers. Finally, the study evaluated the degradation efficiency and reaction time of methylene blue solution. The results are presented in Table 3.

**Table 3:** Degradation efficiency and reaction time of methylene blue solution by Ag_3_PO_4_/gC_3_N_4_ and Ti_3_C_2_/gC_3_N_4_ heterojunction photocatalysts.

Catalyst	Capture agent	Degradation rate/%	Reaction time/min	Dark adsorption removal rate/%	Targeted active species	Proportion of relative contribution of active substances/%
Ag_3_PO_4_/gC_3_N_4_	No capturer	100	15	12 ± 1	/	/
	Isopropyl alcohol	68	50		·OH	32 ± 2
	EDTA-2Na	93	45		h⁺	7 ± 1
	Benzoquinone	68	60		·O₂⁻	32 ± 2
	Silver nitrate	92	20		e⁻	8 ± 1
Ti_3_C_2_/gC_3_N_4_	No capturer	98	50	10 ± 1	/	/
	Isopropyl alcohol	45	80		·OH	54 ± 3
	EDTA-2Na	84	75		h⁺	14 ± 2
	Benzoquinone	52	120		·O₂⁻	46 ± 3
	Silver nitrate	85	60		e⁻	13 ± 2

Note: The data in the table all take methylene blue (MB, 5 mg/L) as the target pollutant. The dark adsorption removal rate is the adsorption equilibrium result after the catalyst was mixed with 5 mg/L methyl blue solution and stirred for 30 minutes under no light conditions. The data is the mean ± standard deviation of three parallel experiments. The symbol “-” indicates the influence of this group of focused capture agents on photocatalysis, and the dark adsorption data were not repeatedly measured.

**Fig 15:**
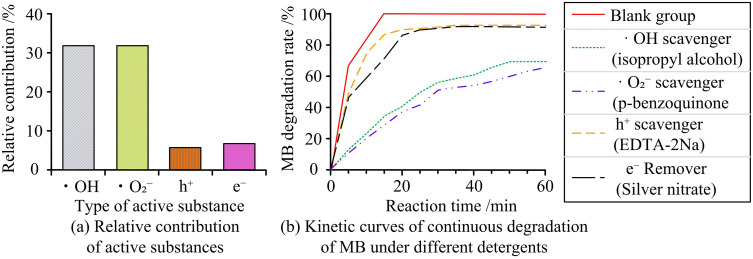
Relative contribution of active species and continuous degradation kinetics curve of MB degraded by Ag₃PO₄/g-C₃N₄ photocatalyst.

As shown in Table 3, the degradation rates of Ag_3_PO_4_/g-C_3_N_4_ and Ti_3_C_2_/g-C_3_N_4_ varied under different scavenger conditions. Without any scavenger, it achieved a maximum degradation rate of 100% within 15 minutes. In comparison, Ti_3_C_2_/g-C_3_N_4_ only reached 98%, and its reaction time was 35 minutes longer. When EDTA-2Na was used as the scavenger, the degradation rates decreased to 93% and 84%, respectively. When AgNO₃ was used, the reaction time for Ag_3_PO_4_/g-C_3_N_4_ was 20 minutes, while Ti_3_C_2_/g-C_3_N_4_ required 60 minutes. When isopropanol (·OH capture agent) or benzoquinone (O_2_^-^· capture agent) was added, the efficiency of Ag_3_PO_4_/g-C_3_N_4_ in degrading MB decreased significantly, while the degradation rate remained at 93% when EDTA-2Na was added, indicating that ·OH and O_2_^-^· were the main active species, and the contribution of h^+^ was weak. This result is consistent with the charge transfer paths of retaining the g-C_3_N_4_ conduction band e⁻ and the Ag_3_PO_4_ valence band h⁺ in the Z-scheme mechanism, eliminating the possibility of traditional type II heterojunctions, and is consistent with the characteristics of active species in the Z-scheme system reported by Li X et al. [23]. These results confirmed that the Z-scheme heterojunction mechanism established by Ag_3_PO_4_/g-C_3_N_4_ efficiently inhibited the recombination of photoinduced electron-hole pairs and facilitated charge carrier separation. The preserved strong oxidative holes allowed the catalyst to maintain high activity even. Moreover, in the Ag_3_PO_4_/g-C_3_N_4_ system without a capture agent, it is 12 ± 1%, while in the Ti_3_C_2_/g-C_3_N_4_ system, it corresponds to 10 ± 1%. This indicates that the former has a slightly stronger ability to adsorb methyl blue in the dark state, which can provide a basic difference reference for photocatalytic degradation. The continuous degradation kinetics and relative contribution of active species are shown in Fig 15.

**Fig 16:**
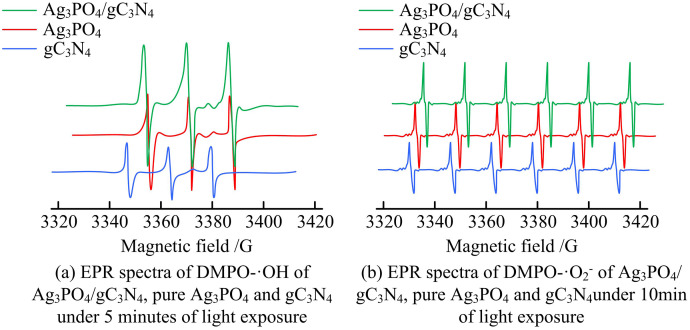
DMPO-·OH and DMPO-·O_2_^-^ EPR spectra of Ag_3_PO_4_/gC_3_N_4_, pure Ag_3_PO_4_ and gC_3_N_4_ under illumination.

As shown in Fig 15a, ·OH and ·O₂ ⁻ have the highest relative contribution to MB degradation, which further confirms that the Z-scheme heterojunction promotes the efficient generation of these two active species. Fig 15b shows the continuous degradation process: the blank group reaches 100% degradation in 15 minutes, while the groups added with ·OH or ·O₂ ⁻ scavengers show significantly slower degradation rates (only 68% degradation in 50–60 minutes), which is consistent with the relative contribution results and verifies the reliability of the quantitative analysis. Subsequently, EPR tests were conducted under the conditions of a 300W xenon lamp, λ ≥ 420 nm, and a DMPO concentration of 50 mmol/ L. The DMPO-·OH and DMPO-·O_2_^-^-EPR spectra of Ag_3_PO_4_/gC_3_N_4_, pure Ag_3_PO_4_, and gC_3_N_4_ under illumination are shown in Fig 16.

It can be seen from Fig 16a that the characteristic peak of the Ag_3_PO_4_/gC_3_N_4_ heterojunction is the most prominent, with a peak intensity higher than that of pure Ag_3_PO_4_ and gC_3_N_4_, indicating that the heterojunction has a stronger ability to generate hydroxyl radicals (·OH). In Figure (b), the Ag_3_PO_4_/gC_3_N_4_ heterojunction shows clear and regular characteristic peaks. Compared with the pure components, its ability to generate superoxide free radicals (·O_2_^-^) is more prominent. This indicates that the Ag_3_PO_4_/gC_3_N_4_ heterojunction can not only efficiently generate ·OH, but also produce a large amount of ·O_2_^-^ under longer light exposure time. The heterojunction structure promotes charge separation and carrier lifetime extension, providing sufficient active species for its photocatalytic performance, and also demonstrates that this heterojunction plays a significant role in photocatalytic reactions. It has a good promoting effect on the generation of different active free radicals. The cyclic degradation performance of Ag_3_PO_4_/g-C_3_N_4_ and the control sample is shown in Table 4.

**Table 4:** Cyclic degradation performance of Ag_3_PO_4_/g-C_3_N_4_ and control samples.

Catalyst	Number of cycles	Degradation rate/%	Reaction time/min	Catalyst recovery rate/%
Ag_3_PO_4_/gC_3_N_4_	1	100	15	95 ± 2
	2	97	16	94 ± 2
	3	95	17	93 ± 1
	4	92	18	92 ± 1
Ag_3_PO_4_	1	96	62	88 ± 3
	2	85	68	82 ± 2
	3	78	75	76 ± 3
	4	72	80	70 ± 2
gC_3_N_4_	1	32	128	96 ± 1
	2	30	132	95 ± 1
	3	29	135	94 ± 2
	4	28	138	93 ± 2

Note: The target pollutant for the cyclic experiment is methylene blue (MB, 5 mg/L), and the reaction conditions are the same as those in Table 3.

As can be seen from Table 4, Ag_3_PO_4_/g-C_3_N_4_ performed exceptionally well, with a degradation rate of 100% in the first cycle and a reaction time of only 15 minutes. After four cycles, the degradation rate remained at 92%, and the reaction time was increased to 18 minutes. Moreover, the recovery rate of the catalyst remained stable at 92%−95%, demonstrating excellent cycling stability. The initial degradation rate of Ag_3_PO_4_ was 96%, but as the number of cycles increased, the degradation rate dropped from 96% to 72%, the reaction time was extended from 62 minutes to 80 minutes, and the recovery rate gradually decreased, with poor stability. The degradation effect of g-C_3_N_4_ is the poorest. The initial degradation rate is only 32%, and it drops to 28% after four cycles. The reaction time is continuously prolonged. Although the recovery rate is relatively high, maintained at 93%−96%, the low degradation rate limits its application. The Z-scheme can retain strongly oxidizing h^+^ to inhibit the photo-corrosion of Ag_3_PO_4_, and the degradation rate still reaches 92% after 4 cycles. This is similar to the conclusion reported by Li X et al. that Z-scheme enhances the stability of Ag₃PO₄ [24]. By comparison, it can be seen that the Ag_3_PO_4_/g-C_3_N_4_ heterojunction effectively enhances the stability and efficiency of the photocatalytic cycle, with significant advantages. The effects of different regeneration methods on the cyclic degradation performance of Ag_3_PO_4_/g-C_3_N_4_ are shown in Table 5.

**Table 5:** Effects of Different regeneration Methods on the cyclic degradation performance of Ag₃PO₄/g-C₃N₄.

Regeneration method	Number of cycles	Degradation rate/%	Reaction time/min	Catalyst recovery rate/%
Deionized water washing	1	100	15	95 ± 2
	2	97	16	94 ± 2
	3	95	17	93 ± 1
	4	92	18	92 ± 1
Wash with 50% ethanol	1	100	15	94 ± 2
	2	95	17	92 ± 2
	3	91	19	90 ± 1
	4	88	20	88 ± 1
Heat treatment at 100°C	1	100	15	95 ± 1
	2	96	16	94 ± 1
	3	93	18	92 ± 2
	4	90	19	91 ± 2

In Table 5, for the catalyst regenerated by deionized water washing, the degradation rate decreased from 100% to 92% after four cycles. The reaction time increased by 3 minutes, and the recovery rate remained stable at 92%−95%, with a gentle performance degradation. When regenerating with 50% ethanol washing, the degradation rate decreased from 100% to 88% with the cycle. The reaction time was extended by 5 minutes, and the performance decline was relatively obvious. After 100°C heat treatment and regeneration, the degradation rate dropped to 90% after 4 cycles. The reaction time was increased by 4 minutes, and the recovery rate was maintained at 91%−95%. Overall, the deionized water washing regeneration method performs better in maintaining the degradation rate, controlling the extension of reaction time, and maintaining the recovery rate of the catalyst. The 50% ethanol washing method may have a certain impact on the structure or surface properties of the catalyst, and the overall effect of 100°C heat treatment is not as good as that of deionized water washing. This result provides data support for the selection of efficient regeneration methods for Ag₃PO₄/g-C₃N₄ catalysts.

## 4. Discussion

The Ag_3_PO_4_/g-C_3_N_4_ developed in this study showed significant advantages in performance analysis. The material prepared in the study has an efficiency advantage of 100% degradation within 15 minutes without a capture agent, which is attributed to the synergistic effect of the Z-scheme charge transfer pathway and the high specific surface area. Ag_3_PO_4_ nanoparticles synthesized via the in situ precipitation approach were homogeneously dispersed across the g-C_3_N_4_ nanosheet surfaces. These particles closely covered the g-C_3_N_4_ substrate without obvious aggregation. This was mainly due to the atomic-level interface binding achieved through the synergistic effect of chemical bonding and physical confinement. These observations aligned with the findings reported by M. Belhaj et al. [25]. From the XRD crystallographic data and IR spectral characterization of g-C_3_N_4_, 10% Ag_3_PO_4_/g-C_3_N_4_, 20% Ag_3_PO_4_/g-C_3_N_4_, 30% Ag_3_PO_4_/g-C_3_N_4_, and pure Ag_3_PO_4_, the signature peaks attributed to Ag_3_PO_4_ demonstrated comparatively weak signals. This indicated that Ag_3_PO_4_ was uniformly dispersed at low loading levels without significant aggregation. This was attributed to the coordination between amino and imino groups in g-C_3_N_4_ and Ag ⁺ , which formed Ag–N bonding sites and provided nucleation points for atomic-level dispersion of Ag_3_PO_4_. Similar results were also obtained by H. Li et al. [26]. From the UV–visible absorption spectra and specific surface area data of Ag_3_PO_4_/g-C_3_N_4_, Ag_3_PO_4_, and g-C_3_N_4_, the specific surface area was 42.45 m^2^·g^-1^. This enhancement resulted from the establishment of a Z-scheme heterojunction through chemical coupling, where new pore structures appeared at the interface, expanding the surface area. The cubic Ag_3_PO_4_ particles dispersed in a quasi-spherical structure. Its surface roughness, together with the two-dimensional sheet-like structure of g-C_3_N_4_, contributed to improved porosity. This structural advantage provided more adsorption and reaction sites for photocatalysis, thus enhancing the catalytic activity. This trend was consistent with the findings of T. Liu et al. in 2024 [27].

Based on the bandgap and light absorption results of different samples, Ag_3_PO_4_ exhibited strong light absorption in the 200–580 nanometer range. The absorption threshold of g-C_3_N_4_ was positioned at approximately 450 nanometers. In contrast to g-C_3_N_4_, the absorption thresholds of the composite photocatalysts exhibited a pronounced shift toward longer wavelengths (bathochromic shift) with increasing Ag_3_PO_4_ loading. The integration of 2D g-C_3_N_4_ nanosheets with Ag_3_PO_4_ particles established a mesoporous network architecture within the samples. This mesoporous framework delivered an enhanced surface area along with increased active site density, thereby promoting pollutant adsorption and interfacial reaction processes throughout the photocatalytic procedure. These advantages came from the Z-scheme heterojunction constructed via in situ growth, which integrated the strengths of Ag_3_PO_4_ and g-C_3_N_4_. The plasmonic resonance effect on the Ag_3_PO_4_ surface improved its visible light capture ability, while the 2D layered structure of g-C_3_N_4_ promoted charge separation by extending the conjugated system. The band bending formed in the heterojunction further broadened the light response range. These results aligned with the conclusions of W. Luo et al. [28]. The photocatalytic degradation results of Rhodamine B showed that the degradation rates of Ti_3_C_2_/g-C_3_N_4_ and Ti_3_C_2_/Ag_3_PO_4_ reached 63% and 82%, respectively, after 133 minutes and 38 minutes. However, their performance was still lower. After 75 minutes, the degradation rate of methylene blue stabilized at over 98%. This was attributed to the joint catalytic activity centers—Ag⁺ vacancies in Ag_3_PO_4_ and nitrogen vacancies in g-C_3_N_4_. Moreover, the tight chemical interface formed by the in-situ growth method reduced charge transfer resistance and improved carrier separation efficiency. These findings were comparable to the observations of R. D. Divedi et al. [29]. Water washing can effectively remove surface adsorbed contaminants without damaging the heterojunction interface, making it the most suitable regeneration method and providing a basis for reducing costs and improving reusability in practical applications [30,31]. The study achieved efficient regeneration of Ag₃PO₄/g-C₃N₄ catalyst by washing with deionized water. This method is consistent with the principle of the “Washing regeneration strategy of phenyl-modified g-C₃N₄/TiO₂ Catalyst” proposed by Porcu S et al. ([30]). Both remove surface adsorbed contaminants through physical cleaning, avoiding the damage of chemical reagents to the heterogeneous interface. However, the reaction time of the studied catalyst was only extended by 3 minutes after water washing, and its stability was superior to that of the material in reference [30]. This was attributed to the atomic-level tight interface constructed by the Ag-N coordination bond, which reduced the detachment of active components during the water washing process. The specific surface area of Ag₃PO₄/g-C₃N₄ is higher than that of pure Ag₃PO₄ and g-C₃N₄. This result is consistent with the findings of Hazra M et al. ([31]) in the 2D-2DPhCN/WS_2_ heterojunction. Both construct mesoporous structures through the composite of two-dimensional materials to expand the specific surface area and increase the number of active sites. However, the Ag₃PO₄/g-C₃N₄ mesoporous pores formed by the in-situ growth method in the study have more uniform pore sizes, which are superior to the pore size distribution of the materials in reference [31], and are more conducive to the rapid diffusion and adsorption of pollutants. The study achieved atomic-level binding of Ag₃PO₄ and g-C₃N₄ through in-situ growth method, which is similar to the interface regulation idea of “phenyl-modified g-C₃N₄/TiO_2_ polymer hybrids” proposed by Dettori R et al. ([32]), both reducing interface defects through chemical coupling. However, [32] relies on polymer modification to enhance interface stability, while the study achieves interface optimization without additives through Ag-N coordination bonds, which is more in line with the concept of green catalysis and is superior to the material in reference [32] in terms of the efficiency of visible light degradation of MB. By regulating the molar ratio of reactants and calcination temperature, the study achieved the directional growth of Ag_3_PO_4_ between g-C_3_N_4_ layers, forming heterojunction structures with interlaced thicknesses, providing a new paradigm for atomic-level interfacial regulation. Compared with Wang et al.’s study on the SnFe₂O₄/ZnFe₂O₄ S-scheme heterojunction, the Z-scheme electron transfer pathway proposed in this study detected the characteristic signals of ·OH and ·O₂⁻ via electron paramagnetic resonance (EPR) spectroscopy, with significantly enhanced signal intensities [33]. This result is consistent with the finding from the radical trapping experiments in this study that “degradation is dominated by ·OH and ·O₂⁻”, confirming that the Z-scheme mechanism enables efficient generation of reactive oxygen species (ROS). In comparison to Zhao et al.’s research on PbTiO₃ ferroelectric photocatalysis, the interface potential difference of the heterojunction measured in this study using Kelvin probe force microscopy (KPFM) was 0.32 eV. This value is close to the “0.35 eV interface potential difference between Ag₃PO₄ and g-C₃N₄” calculated from the Mott-Schottky curves in this work, which further reveals the energy driving force for electron transfer in the proposed Z-scheme mechanism [34]. The experiment confirmed the Z-type electron transfer path of Ag_3_PO_4_/g-C_3_N_4_, providing a reference for the mechanism analysis of similar catalysts. It addresses the problem of photocorrosion of traditional Ag_3_PO_4_ (the degradation rate of Ag_3_PO_4_/g-C_3_N_4_ still reaches 92% after four cycles, while that of pure Ag_3_PO_4_ is only 72%), providing a practical solution for the design of efficient and stable photocatalysts and reducing the cost of industrial applications. Compared with the Fe_2_O_3_/g-C_3_N_4_/TiO_2_ heterojunctions prepared by Y. Nien et al. through the dual-jet electrospinning method, both constructed heterojunctions with g-C3N4 as the substrate to enhance photocatalytic performance. However, the in-situ growth method studied achieved atomic-level tight binding of Ag_3_PO_4_ and g-C_3_N_4_ through Ag-N coordination bonds. The density of interface defects is lower, while in the study by Y. Nien et al., the physically combined heterojunction had a loose interface, and the charge transfer resistance significantly increases after long-term cycling, resulting in a considerable decline in photocatalytic efficiency. Compared with the CsBi_3_I_10_/TiO_2_ II heterojunction constructed by T. Liu et al., although it improves the carrier separation efficiency through band matching, at the expense of REDOX capacity, the degradation rate of Rhodamine B is only 82%. The Z-shaped heterojunction studied can simultaneously retain the strong oxidizing holes in the Ag_3_PO_4_ valence band and the strong reducing electrons in the g-C_3_N_4_ conduction band, which effectively inhibits photocorrosion. In addition, M. Belhaj et al. pointed out that the interface quality of heterojunctions is the core factor affecting photocatalytic performance. The study confirmed the formation of Ag-O-C bonds through XPS, further verifying the advancement of in-situ growth methods in interface regulation. The reaction rate far exceeded the light response speed of polymer/ZnO heterojunctions in the research of M. Belhaj scholars. It provides a new paradigm for the design of efficient and stable photocatalysts.

This study made contributions in several aspects. First, in material design, the directional growth of Ag_3_PO_4_ nanoparticles between g-C_3_N_4_ layers was achieved by precisely controlling the molar ratio of reactants and calcination temperature, forming a heterojunction with an interlaced thickness structure. Second, in terms of mechanism, experiments confirmed the Z-scheme electron transfer pathway in the Ag_3_PO_4_/g-C_3_N_4_. Third, in application, the composite achieved efficient degradation of methylene blue and solved the problem of light corrosion in traditional Ag_3_PO_4_, providing a new paradigm for designing highly efficient and stable photocatalysts.

## 5. Conclusion

To address the problems of poor interfacial contact, low charge separation efficiency, and limited stability in current heterojunction fabrication methods, this study put forward an in situ growth method to prepare an Ag_3_PO_4_/g-C_3_N_4_. The goal was to develop a highly stable heterojunction to overcome the issues of weak interfacial bonding and photocorrosion seen in conventional approaches. The results showed that Ag_3_PO_4_/g-C_3_N_4_ had a narrower band gap than g-C_3_N_4_, indicating improved photogenerated charge carrier generation and higher photocatalytic efficiency. The weak characteristic peaks of Ag_3_PO_4_ suggested that it was uniformly dispersed at low loading levels without obvious agglomeration. SEM and TEM images of the heterojunction displayed a tightly connected interface with uniformly distributed particles, contributing to enhanced photocatalytic performance. The remarkable similarity between the XRD diffraction patterns of Ag_3_PO_4_/g-C_3_N_4_ composites and pristine g-C_3_N_4_, with retention of characteristic peaks and minimal structural distortion, further substantiated the homogeneous dispersion and uniform loading of Ag_3_PO_4_ nanoparticles across the g-C_3_N_4_ surface. This crystallographic consistency indicates successful interfacial integration without compromising the structural integrity of either semiconductor component. In summary, the strategically fabricated Ag_3_PO_4_/g-C_3_N_4_ significantly enhanced the separation efficiency through the synergistic effects of intimate interfacial contact and optimized electronic band structure alignment. The formation of internal electric fields at the heterojunction interface collectively contributed to superior photocatalytic performance and reduced electron-hole pair recombination compared to individual semiconductor materials. However, it still suffers from issues such as Ag⁺ leaching due to photocorrosion and limited adaptability in complex water environments. In the future, constructing a ternary Z-scheme system will further enhance charge separation efficiency and improve its overall stability and performance in practical applications.

## Supporting information

S1 File Minimal Data Set Definition. (DOC)
