# Supplementary material for: Fabrication of Ag3PO4/g-C3N4 heterojunction photocatalyst via in-situ growth and its photocatalytic performance
Source: PLoS One. 2025 Dec 9;20(12):e0337123. doi: 10.1371/journal.pone.0337123 (PMC12688128; doi:10.1371/journal.pone.0337123)
Supplement: S1 File — (DOC) [file pone.0337123.s001.doc]

**Figure 6 XRD patterns and infrared spectra**

**Figure 6(a)**

| 2θ /° | Strength | | | | |
| --- | --- | --- | --- | --- | --- |
|  | Ag3PO4 | 10%Ag3PO4/gC3N4 | 20%Ag3PO4/gC3N4 | 30%Ag3PO4/gC3N4 | gC3N4 |
| 10 | 788 | 1267 | 1568 | 1825 | 2258 |
| 20 | 726 | 1239 | 1593 | 1892 | 2249 |
| 30 | 749 | 1247 | 1487 | 1873 | 2186 |
| 40 | 753 | 1193 | 1506 | 1726 | 2094 |
| 50 | 681 | 1346 | 1672 | 1943 | 2203 |
| 60 | 629 | 1237 | 1509 | 1862 | 2147 |
| 70 | 646 | 1168 | 1448 | 1763 | 2004 |

**Figure 6(b)**

| Wave n umber /cm^-1^ | Transmittance /% | | | | |
| --- | --- | --- | --- | --- | --- |
|  | gC3N4 | 10%Ag3PO4/gC3N4 | 20%Ag3PO4/gC3N4 | 30%Ag3PO4/gC3N4 | Ag3PO4 |
| 500 | 90.63 | 37.48 | 52.84 | 75.91 | 18.64 |
| 1500 | 90.58 | 29.43 | 50.72 | 70.46 | 16.13 |
| 2500 | 90.82 | 37.98 | 53.67 | 52.31 | 19.73 |
| 3500 | 90.91 | 38.63 | 49.71 | 51.48 | 17.25 |

**Figure 7 TGA curves of Ti_3_C_2_/g-C_3_N_4_ and Ag_3_PO_4_/g-C_3_N_4_ heterojunctions**

| Temperature /℃ | Weight percentage /% | |
| --- | --- | --- |
|  | Ti_3_C_2_/gC3N4 | Ag3PO4/gC3N4 |
| 100 | 99.28 | 99.78 |
| 200 | 98.91 | 99.69 |
| 300 | 98.47 | 99.42 |
| 400 | 98.37 | 99.34 |
| 500 | 98.26 | 99.45 |
| 600 | 98.29 | 99.32 |
| 700 | 98.21 | 99.25 |

**Figure 8 UV-visible optical spectra and surface area properties results**

**Figure 8(a)**

| Relative pressure /(*p*/*p*_0_) | Adsorption capacity /(cm^3^·g^-1^) | | |
| --- | --- | --- | --- |
|  | Ag3PO4 | gC3N4 | Ag3PO4/gC3N4 |
| 0.2 | 0.35 | 8.47 | 9.14 |
| 0.4 | 0.56 | 8.96 | 15.47 |
| 0.6 | 0.74 | 16.42 | 20.14 |
| 0.8 | 0.61 | 28.79 | 45.23 |
| 1.0 | 0.47 | 87.61 | 99.71 |

**Figure 8(b)**

| Sample | Specific surface area /(m^2^·g^-1^) |
| --- | --- |
| Ag3PO4 | 4.69 |
| gC3N4 | 33.82 |
| Ag3PO4/gC3N4 | 42.45 |

**Figure 9 Bandgap and absorbance results**

**Figure 9(a)**

| Binding energy (eV) | Bandgap width (eV) | | |
| --- | --- | --- | --- |
|  | gC3N4 | 20%Ag3PO4/gC3N4 | Ag3PO4 |
| 2.0 | 0.68 | 0.75 | 1.15 |
| 2.5 | 0.76 | 1.14 | 1.52 |
| 3.0 | 1.23 | 1.65 | 2.17 |
| 3.5 | 1.74 | 1.82 | 2.23 |
| 4.0 | 1.97 | 2.02 | 2.34 |

**Figure 9(b)**

| Wavelength (nm) | Absorbance | | | | |
| --- | --- | --- | --- | --- | --- |
|  | Ag_3_PO_4_ | g-C_3_N_4_ | 10%Ag_3_PO_4_/g-C_3_N_4_ | 20%Ag3PO4/gC3N4 | 30%Ag_3_PO_4_/g-C_3_N_4_ |
| 200 | 0.52 | 0.38 | 0.29 | 0.17 | 0.37 |
| 400 | 1.35 | 1.09 | 0.97 | 0.88 | 1.06 |
| 600 | 0.91 | 0.71 | 0.54 | 0.49 | 0.58 |
| 800 | 0.73 | 0.66 | 0.36 | 0.32 | 0.34 |

**Figure 10 photocatalytic removal of Rhodamine B via visible light irradiation**

| Time /min | C/C_0_ | | | | | |
| --- | --- | --- | --- | --- | --- | --- |
|  | Ti_3_C_2_ | Ag3PO4 | gC3N4 | Ti_3_C_2_/g-C_3_N_4_ | Ti_3_C_2_/Ag_3_PO_4_ | Ag_3_PO_4_/g-C_3_N_4_ |
| -20 | 1.02 | 0.97 | 0.98 | 1.02 | 1.01 | 1.00 |
| 0 | 0.95 | 0.76 | 0.97 | 1.01 | 0.98 | 0.91 |
| 20 | 0.92 | 0.53 | 0.86 | 0.98 | 0.91 | 0.71 |
| 40 | 0.93 | 0.28 | 0.87 | 0.89 | 0.78 | 0.26 |
| 60 | 0.91 | 0.13 | 0.83 | 0.86 | 0.71 | 0.11 |
| 80 | 0.92 | 0.07 | 0.78 | 0.83 | 0.57 | 0.06 |
| 100 | 0.94 | 0.06 | 0.79 | 0.82 | 0.51 | 0.07 |
| 120 | 0.92 | 0.05 | 0.73 | 0.78 | 0.49 | 0.07 |
| 140 | 0.89 | 0.03 | 0.71 | 0.76 | 0.47 | 0.06 |

**Figure 11 PL spectra, EIS, and TPR curves**

**Figure 11(a)**

| Wavelength /nm | PL intensity /arb.units | | |
| --- | --- | --- | --- |
|  | g-C_3_N_4_ | Ag_3_PO_4_/g-C_3_N_4_ | Ag_3_PO_4_ |
| 400 | 16.84 | 16.85 | 16.84 |
| 450 | 185.13 | 96.78 | 16.85 |
| 500 | 225.84 | 178.94 | 16.87 |
| 550 | 37.81 | 37.82 | 16.85 |
| 600 | 29.41 | 29.37 | 16.84 |

**Figure 11(b)**

| Z〞/Ω | -Z〞/Ω | | |
| --- | --- | --- | --- |
|  | g-C_3_N_4_ | Ag_3_PO_4_/g-C_3_N_4_ | Ag_3_PO_4_ |
| 1000 | 2896 | 1567 | 497 |
| 2000 | 3874 | 2391 | / |
| 3000 | / | 2783 | / |
| 4000 | / | 3274 | / |

**Figure 11(c)**

| Time /s | Photocurrent density /(mA·cm^-2^) | | |
| --- | --- | --- | --- |
|  | Ag_3_PO_4_/g-C_3_N_4_ | g-C3N4 | Ag3PO4 |
| 60 | 0.057 | 0.047 | 0.012 |
| 120 | 0.064 | 0.054 | 0.017 |
| 180 | 0.082 | 0.048 | 0.016 |
| 240 | 0.058 | 0.025 | 0.008 |
